# Access to preventive sexual and reproductive health care for women from refugee-like backgrounds: a systematic review

**DOI:** 10.1186/s12889-022-12576-4

**Published:** 2022-02-27

**Authors:** Natasha Davidson, Karin Hammarberg, Lorena Romero, Jane Fisher

**Affiliations:** 1 Global and Women’s Health, School of Public Health and Preventive Medicine, Monash University Faculty of Medicine Nursing and Health Sciences, Melbourne, VIC Australia; 2 The Ian Potter Library, The Alfred Hospital, Melbourne, Victoria Australia

**Keywords:** Refugees, Women, Sexual and reproductive health, Health care providers, Access

## Abstract

**Background:**

Globally, the number of forcibly displaced women is growing. Refugee and displaced women have poorer health outcomes compared to migrant and host country populations. Conflict, persecution, violence or natural disasters and under-resourced health systems in their country of origin contribute to displacement experiences of refugee and displaced women. Poor health outcomes are further exacerbated by the migration journey and challenging resettlement in host countries. Preventive sexual and reproductive health (SRH) needs of refugee and displaced women are poorly understood. The aim was to synthesise the evidence about access to preventive SRH care of refugee and displaced women.

**Methods:**

A systematic review of qualitative, quantitative and mixed methods studies of women aged 18 to 64 years and health care providers' (HCPs’) perspectives on barriers to and enablers of SRH care was undertaken. The search strategy was registered with PROSPERO in advance of the search (ID CRD42020173039). The MEDLINE, PsycINFO, Embase, CINAHL, and Global health databases were searched for peer-reviewed publications published any date up to 30th April 2020. Three authors performed full text screening independently. Publications were reviewed and assessed for quality. Study findings were thematically extracted and reported in a narrative synthesis. Reporting of the review followed the Preferred Reporting Items for Systematic Reviews and Meta-Analyses recommendations.

**Results:**

The search yielded 4083 results, of which 28 papers reporting 28 studies met inclusion criteria. Most related to contraception and cervical or breast cancer screening. Three main themes and ten subthemes relating to SRH care access were identified: interpersonal and patient encounter factors (including knowledge, awareness, perceived need for and use of preventive SRH care; language and communication barriers), health system factors (including HCPs discrimination and lack of quality health resources; financial barriers and unmet need; HCP characteristics; health system navigation) and sociocultural factors and the refugee experience (including family influence; religious and cultural factors).

**Conclusions:**

Implications for clinical practice and policy include giving women the option of seeing women HCPs, increasing the scope of practice for HCPs, ensuring adequate time is available during consultations to listen and develop refugee and displaced women’s trust and confidence, strengthening education for refugee and displaced women unfamiliar with preventive care and refining HCPs’ and interpreters’ cultural competency. More research is needed on HCPs’ views regarding care for refugee and displaced women.

**Supplementary Information:**

The online version contains supplementary material available at 10.1186/s12889-022-12576-4.

## Background

Globally, the number of people who are forcibly displaced both within countries and across borders as a result of conflict, persecution, violence or natural disasters has grown by over 50% in the last 10 years. In 2009, 43.3 million people were forcibly displaced, increasing to 79.5 million at the end of 2019 [1]. Of those, 45.7 million comprise internally displaced people, 26 million refugees and 4.2 million asylum-seekers [1]. Forcibly displaced people include those who have meet the United Nations criteria for being a refugee [2], those seeking asylum who are not yet granted refugee status and internally displaced people who have fled their region of origin within their country but have not crossed an international border. In general, refugee and displaced people with past and current migration experiences are considered vulnerable members of the community. The experiences and potential vulnerabilities of women and girls differ significantly from those of men and boys. Women are often afforded lower social status than men which places them in a position of dependency to men. Lack of educational opportunities make it more difficult for women to access decision-making positions and safe employment opportunities. At least half the forcibly displaced people are women and girls [3] with many living for protracted periods in refugee camps in poor conditions [4]. We acknowledge the importance of person-first language but in the interests of brevity, throughout this paper we refer to women from refugee-like backgrounds as “refugee and displaced women”. This term signifies the context of women’s refugee-like backgrounds and experiences. By definition refugee and displaced women have fled their country or region of origin. The refugee experience places these women in situations which create vulnerability.

Pre-migration experiences caused by violence, torture, rape or witnessing the torture or killing of family or friends are associated with poor psychological and physical health outcomes [5]. Postmigration stress also contributes to poor general health, particularly in refugee and displaced women [6]. Most refugee and displaced women have not voluntarily chosen to leave their country of origin, often depart at short notice, have lengthy journeys within their own country or crossing international borders. They may be separated from family members in transit or at the time of resettlement, have reduced social support systems, be survivors of torture and have lost most of their material possessions, wealth and status [7]. Further, access to ongoing, familiar health services is lost [8]. In their systematic review of young women in Africa, Ivanovna and colleagues [9] concluded that access to and availability of sexual and reproductive health (SRH) care is often limited in low-income country humanitarian settings. As a result, refugee and displaced women are at risk of adverse health outcomes such as unintended pregnancy due to a lack of access to contraception [10, 11], lack of access to abortion services [12], sexually transmitted infections [13] and sexual and gender-based violence [14].

Universal publicly available access to SRH care has been recognized by the World Health Organisation as a priority in global health [15]. It is also of key importance in Sustainable Development Goal 3 which seeks to ensure good health and wellbeing for all and Sustainable Development Goal 5 which seeks to achieve gender equity [16]. In World Bank classified [17] low-income countries however, women’s health services, particularly preventive SRH care including contraception, cervical and breast cancer screening and human papilloma virus (HPV) vaccination is often not available or not of a quality that meets the World Health Organisation framework for human rights standards [18]. A fact further supported at the International Conference on Human Rights in 2013 [19]. Poor quality relates to lack of universal access to SRH care and scarce human and financial resources [20] in addition to lack of patient centred care [21] and respectful, effective and efficient communication [22]. The concept of an Essential Package of Health Services was initiated by World Health Organisation in 2014 to progress universal access to SRH. Its aim was to provide priority health services for vulnerable populations in fragile settings, where needs often exceed available resources [15]. The Essential Package of Health Services across most low- and middle-income countries excludes many SRH services [23]. An analysis based on low- and middle-income countries’ health services [20] showed almost all countries included maternal health care and some had SRH care for family planning and sexually transmitted infection/human immunodeficiency virus prevention and management. However, the majority did not mention infertility, or screening for cervical and breast cancers. By contrast, most women in high-income countries have free or low cost universal SRH access in primary health care including screening for reproductive cancers [24]. The poor quality of SRH care but also a lack of available services, leads to low utilisation of these services [25] further contributing to barriers refugee and displaced women may experience in accessing care [26]. In 2004, a global evaluation of reproductive health services for refugees and internally displaced people concluded that most people affected by conflict lack adequate SRH care. Refugee and displaced women have been overlooked in humanitarian and in transit low-income country settings and consequently have unmet SRH needs and poor SRH outcomes [27].

Following resettlement refugee and displaced women’s use of primary health care is limited. A systematic review by Hadgkiss and Renzaho [28] examining asylum seekers residing in the community in high-income countries found they had higher tertiary level health service use but lower preventive health service use than the host population. Annual hospitalisation rates among asylum seekers in the Netherlands varied from 12 to 20% compared with 7% in the general population. With regard to use of preventive health services, 25% of asylum seekers had undergone a cervical pap screening test compared with 62% in the host population [28]. Similarly, Sarría-Santamera and her colleagues compared use of health services between populations and found over-use of emergency services and under-use of preventive care services among immigrants and refugees compared to host populations [29].

To date research on refugee and migrant women’s access to SRH care has mainly focused on pregnancy, childbirth and post-partum health care [30–35]. Others have focused on people with refugee-like backgrounds experiences in general practice [36, 37]. Few systematic reviews on the topic of SRH disaggregate findings pertaining to refugees, internally displaced people and asylum seekers from those of other immigrants [35, 38]. Refugee and displaced women have particular needs when engaging with the SRH care [39].

Primary HCPs are key to ensuring SRH care needs are meet, yet understanding of HCPs’ perspectives on refugee and displaced women’s access to care is limited. One systematic review of qualitative studies explored challenges and facilitators for HCPs providing general primary healthcare for refugees and asylum seekers in high-income countries [40]. This review identified multiple barriers experienced by HCPs in providing care for refugees and asylum seekers. Factors related to HCPs competency and responsiveness can contribute to the underutilisation of SRH care by refugee and displaced women [41]. Brandenberger and colleagues [38] go one step further and identify the need for comprehensive training of HCPs to understand the specific requirements of migrants and refugees.

There is a gap in the literature that explicitly addresses access to preventive SRH care outside pregnancy and maternal health among refugee, internally displaced and asylum seeker populations. It is not known what the experiences of access to preventive SRH care and provision of care are for refugee and displaced women nor the views of HCPs delivering care to this group. The aim of this review was to synthesize the evidence on barriers to and enablers of access to preventive SRH care from the perspectives of refugee and displaced women and their health care providers.

## Methods

The systematic review was registered with PROSPERO database in advance of the search (ID CRD42020173039). Reporting of the review followed the Preferred Reporting Items for Systematic Reviews and Meta-Analysis recommendations [42].

### Selection criteria

#### Inclusion and exclusion criteria

The inclusion criteria were studies that have been peer-reviewed, used qualitative, quantitative or mixed methods and have investigated barriers to or enablers of access to SRH care from the perspective of refugee and displaced women defined as refugees, asylum seekers and internally displaced people; or HCPs’ views about providing SRH care to refugee and displaced women in primary health care settings. Exclusion criteria were investigations of maternity or obstetric care (Additional File 1).

### Search strategy

The search strategy was devised by ND with the assistance of a specialist information analyst, LR. The MEDLINE, EMBASE, PsycINFO, CINAHL and GLOBAL HEALTH databases were searched for peer reviewed papers published any date up to 30th April 2020. The search strategy was based on the Sample, Phenomenon of Interest, Design, Evaluation, Research type (SPIDER) tool [43] to optimise identification of relevant articles. The detailed search strategy is documented in Additional file 2.

Search limits included: English language and women aged 18 to 64 years. No date or country of setting limits were applied. The included articles’ reference lists were hand-searched for additional relevant articles. Articles identified in the search were exported to EndNote X9. After removal of duplicates the remaining articles were exported to Covidence Systematic Review Management Platform [44].

### Selection

Titles and abstracts were screened for relevance by ND and articles that did not meet inclusion criteria were removed. Full text of the remaining articles were reviewed independently in Covidence [44] by ND, KH and JF. Any disagreements were resolved through discussion to come to a final decision.

### Data extraction

Study characteristics were extracted by ND using a pre-set proforma in Microsoft Excel. Data were extracted on the following key characteristics: author, title, year published, country of study, study aim, theoretical framework, study design, sampling technique, participant characteristics (including age group, country of origin, migration category and time since arrival), sample size, data collection method, data analysis, outcome measures, SRH topic covered, and key findings.

### Quality assessment

Study quality was assessed using the Kmet Standard Quality Assessment Criteria for Evaluating Primary Research framework for appraisal tool [45]. The Kmet tool provides a systematic reproducible and quantitative means of appraising qualitative and quantitative studies. Mixed-methods studies were appraised using both the qualitative and quantitative quality assessment criteria. In addition, for all studies, evidence of human research ethics committee approval was scored 0 for ‘no ethics approval’ and 1 for ‘has ethics approval’ approved by a formally constituted ethics committee.

### Data synthesis

Narrative synthesis is a systematic approach to searching for and quality appraising evidence. In systematic reviews it is used to explore relationships within and between study findings. Narrative synthesis is an area of emerging research in the field of systematic reviews, however there are broad guidelines which have been followed to guide this review process [46]. This method was reported because characteristics of the study designs and outcomes were too diverse to yield a meaningful summary of findings using a meta-analysis.

The main findings and conclusions were grouped and coded inductively into descriptive themes that emerged from the data within the two categories of “barriers to” and “enablers of” access to SRH care, as defined by the review aim. Findings were only coded if they related to barriers to or enablers of SRH care. Data were further grouped into the SRH topic. Findings were coded to iteratively develop and refine descriptive themes, with each study able to contribute new themes. Following the organisation of these descriptive themes, categorisation and generation of higher-level analytical themes were devised. Quantitative data were described separately and used to complement or refute the qualitative evidence.

## Results

**Fig. 1:**
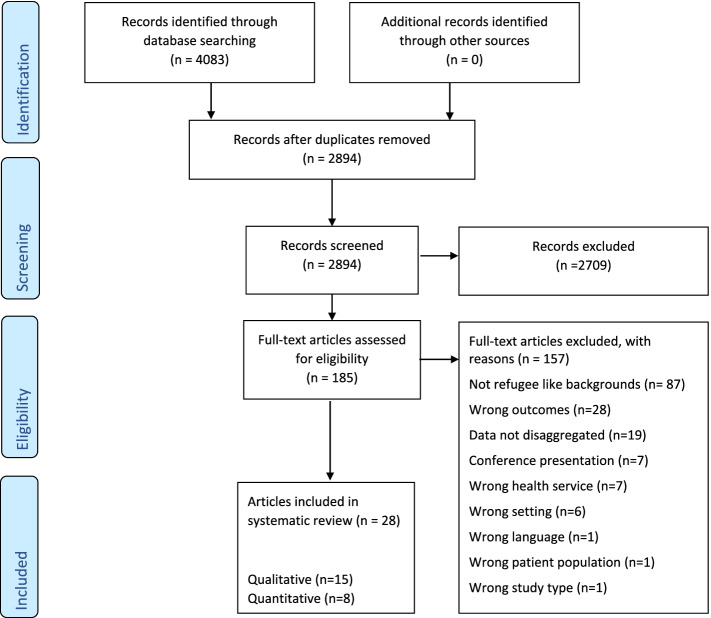
Flow diagram showing the process of study selection (adapted from [42])

Systematic database searches yielded 4083 articles. 28 studies were included in the review (Fig. 1).

### Study characteristics

Study characteristics and main findings are shown in Tables 1 and 2.

**Table 1:** Characteristics of qualitative studies and main findings

Author, (Year); HC; CO	Stated study objectives	Recruitment method; Setting; Data collection method	Sample size; Classification; Age range; Years since arrival	Outcome measures	Data analysis	Main results; Conclusions	Quality score
SRH topic: Family planning (FP)							
Morrison (2000) [47]; HC Thailand; CO Cambodia	Assess the need for contraception among women in Khao Phlu refugee camp and barriers to obtaining and using contraception	Walking through the camps women randomly asked to participate; Khao Phlu refugee camp, Thailand maternal and child health centre; in-depth individual interviews	*n* = 77; refugees; 17–45 yrs. (mean 33); yrs. since arrival N/S	Contraceptive knowledge and beliefs about FP practices	N/S	Barriers to contraceptive use: • uncomfortable seeking contraception, • logistics - heavy rains during the monsoon season prevented travel to health centre, • facilities too far from their section in the camp, • providers’ unwillingness to prescribe contraception - women needed approval from their husbands, • importance placed on marriage and fidelity, Beliefs, cultural norms and misinformation pre-existing barriers for contraceptive use. Stress of refugee experiences may intensify these barriers.	.75
Gurnah et al. (2011) [48]; HC USA; CO Somalia	Exploring the reproductive health care experiences of a marginalized group: the Somali Bantu women in Hartford, Connecticut	Snowball sampling and networking; Hartford; 1 FGD	*n* = 10; refugees; 22–45 yrs. (mean 34); yrs. since arrival mean 4.5 (2.5–5 yrs)	Reproductive health care needs, utilisation and beliefs	Thematic analysis using grounded theory	Barriers to care: • shyness discussing reproductive health with interpreters, • unwillingness to speak to a man about reproductive health problems, • Language Line interpreter did not understand the women, • cultural deference to authority figures - self-advocacy not accepted, • religious prohibitions on contraceptive use, • women chose to forgo care rather than discuss reproductive health topics with a male provider, • desired but limited scope for decision making. A lack of cultural fluency between women and providers.	.65
Cherri et al. (2017) [49]; HC Lebanon; CO Syria	Assess Syrian Refugee Women’s SRH care needs and behaviour and marriage perceptions, to improve services and their acceptability	Convenience sample, women attending clinic or living in informal tented settlements; Governorates of Lebanon- BML, South, North, Bekaa; 11 semi structured FGDs	*n* = 108; refugees; 19–24 yrs. (22.2%) 25–35 yrs. (48.2%) 36–49 yrs. (19.4%); yrs. since arrival 1–6	Perceptions and practices relating to marriage and contraceptive use	Thematic analysis	Perceptions and behaviours relating to contraceptive use: • cost a main barrier to contraceptive use with some women unaware of free services, • OCP (oral contraceptive pill) and intrauterine device most commonly used contraceptives, • rhythm and withdrawal methods perceived as the best and most harmless approaches to birth control, • excess bleeding, menstrual irregularities, back pain, and depression, anger, headache, loss of temper, anxiety, hypertension, and hormonal irregularities were perceived side effects of contraception, • husband’s refusal and family’s interference prevented women from using a contraceptive. • religion was not a barrier to use. Conflict partially changes behaviour and perceptions around contraceptive use.	.70
Kabakkian-Khasholian et al. (2017) [50] HC Lebanon; CO Syria	Explore the perspectives on fertility behaviour and service utilisation of refugee Syrian women and service providers in Al-Marj town in West Bekaa, Lebanon.	Purposive sampling; Al-Marj in West Bekaa; 12 FGDs	*n* = 84; refugees but not officially recognised in Lebanon; 18–45 yrs.; yrs. since arrival N/S	Contraceptive use, decision-making regarding number of children before/after displacement and experiences with health services.	Thematic analysis	Contraceptive decision making: • awareness of dominant role of the male partner in the fertility and FP decision-making, • new roles emerged as a result of displacement. Perceptions about contraceptive needs and provision; • discriminatory treatment received at health facilities, • lack of women providers, • high cost.	.95
Tanabe et al. (2017) [51] HC Bangladesh Djibouti, Jordan, Kenya Malaysia, Uganda; CO Myanmar, Iraq, Democratic Republic of Congo, Somalia	Examine the barriers and challenges at the community and health facility levels that hinder uptake of contraceptives	Purposively selected; Camps, settlements and urban areas in 6 host countries; 12 FGDs and in-depth interviews	*n* = 65 (FGDs) *n* = 23 (interviews); refugees; 20–49 yrs.; yrs. since arrival N/S	Attitudes towards and barriers to contraceptive uptake	N/S	FP services accessibility and barriers to use: • distant service delivery points, • cost of transport to access services, • lack of knowledge about different types of methods, especially emergency contraception, • misinformation and misconceptions, • religious, cultural and language barriers and social stigma, • opposition from husbands, • provider biases - discrimination against refugees, Quality of available services impacted willingness to obtain contraceptives: • lack of adherence to standard precautions, • lack of cleanliness, • long wait times, • limited privacy and confidentiality, Need to scale up FP services in acute emergency and protracted crises humanitarian settings.	.60
West et al. (2017) [52]; HC Jordan; CO Syria	Explore factors that facilitate or limit use of FP services in a Syrian refugee camp in Jordan	Snowball technique; refugee camp in Jordan participants’ tents or caravans; semi structured interviews	*n* = 16; refugees; 18–43 yrs.; yrs. since arrival N/S	Awareness of FP methods, use of FP methods, access to services, service quality, barriers to use, potential improvements.	Frame-work analysis	Awareness of FP methods and services: • knowledge of general health services but poor awareness about FP services limited access to contraceptives. Utilisation and accessibility of FP services: • FP counselling or contraceptive services rarely used, • traditional cultural attitudes towards fertility limited young participants’ uptake of FP, • staff and health services overburdened. Quality of services: • staff conduct seen as inappropriate, • women wanted examinations and consults to be more respectful.	.70
Agbemenu et al. (2018) [53]; HC USA; CO Somalia	Explore the reproductive health decision-making, FP and care during pregnancy and childbirth of Somali Bantu women in Buffalo	Convenience sample via snowball technique; Somali Bantu Community Organization office; 5 semi-structured FGDs	*n* = 30; refugees; 18–35 yrs. (40%) 36–55 (47%) 55+ (10%); yrs. since arrival < 10, (20%) 10–19, (73%) 20+, (3%)	FP decision-making and source of reproductive health education	Content analysis	Barriers to care: • husbands’ resistant to birth control, • husbands have final decision on contraceptive use, • cultural taboo against saying no more children, • natural methods are preferred (e.g. breast feeding) compared with OCPs, • OCPs undesirable due to side effects e.g. consequent inability to get pregnant leading to separation and divorce. Knowledge about reproductive health modalities despite low literacy and limited education.	.85
Ghebreyesus et al. (2020) [54]; Host state Israel; CO Eritrea	Gain a deeper understanding of structural barriers to contraceptive care seeking by Eritrean asylum seekers in Israel	Convenience and snowball sampling; Israeli non-government organisation health facility; 4 FGDs and in-depth interviews	*n =* ~ 32 (FGDs) *n =* 6 (interviews); asylum seekers; 21–30 yrs.; yrs. since arrival N/S	Knowledge of FP and contraception methods, barriers to contraceptive care seeking and vulnerability to unwanted pregnancies	Open, focused and axial coding	Barriers to contraceptive use: • distance to health facilities, • traveling hours by public transport is challenging, • inability to communicate. Limited healthcare resources: • limited time available per patient, • contraceptive product shortages, • navigating a fragmented and complex health system particularly challenging for contraceptive care, • Ministry of Health sites not offering FP services Cost of contraceptive services: • Eritreans excluded from public health insurance, • precarious low-wage employment makes contraceptive care prohibitive, Low standard of care in private clinics: • private gynaecologists offered services but provided incorrect information and prescriptions and did not examine patients prior to administering contraceptives, • women felt discriminated against by providers.	.90
Royer et al. (2020) [55]; HC USA; CO Somalia, Congo.	Evaluate FP knowledge, attitudes and practices (KAP) among refugee women post-resettlement to the USA	Snowball sampling; community centres; 6 FGDs	*n* = 66; refugees; 18–68 yrs.; yrs. since arrival N/S	KAPs regarding reproductive health concerns, access and barriers to care, FP conceptualisation and contraceptive method acceptability	Modified grounded theory	FP KAP: • perceived community preference for many children due to high rates of infant and child mortality, • lack of resources and personal isolation [in USA] changed fertility desires and desired number of children, • FP used to delay first child, birth spacing, birth limiting but depended on method acceptability, partner/family influence, • breastfeeding preferred method of FP as religion permits, • contraception for birth spacing accepted religious practice. Prior to resettlement, men made the final decision about FP but post-resettlement some women reported more equal relationships. Refugee manifestations of FP KAP involve a balance between retention of native culture and incorporation of host country norms.	.85
Zhang et al. (2020) [56]; HC USA; CO Somalia	Exploration of Somali refugee women’s knowledge, attitudes, and experiences with FP and contraception in the USA. Identify barriers and facilitators to contraceptive use	Purposive snowball sampling; King County community centres, participants’ homes; semi structured FGDs	*n* = 53; refugees; < 20- > 35 yrs., (mean 32); yrs. since arrival majority > 10 (mean 15)	Knowledge, attitudes and experiences with birth spacing and contraception	Grounded theory	Barriers to care: • large families valued from a religious and cultural standpoint, • modern contraception not discussed in Somali culture as pre-marital sex is stigmatised, • negative beliefs and fear of side effects regarding OCPs, • belief that modern contraceptives cause infertility and health concerns such as autism and down-syndrome. In contrast to a westernised approach to FP that focuses on individual autonomy, intentional choices and planned behaviours, religious fatalism strongly influences Somali women’s FP attitudes and contraceptive behaviours.	.90
SRH topic: cervical cancer screening							
Haworth, et al. (2014) [57] HC USA; CO Bhutan	Assess KAP for cervical cancer and its screening modalities among Bhutanese refugee women	Convenience sampling; Nebraska community venues and residences; 2 FGDs	*n* = 27; refugees; 19 to 60 + yrs.; yrs. since arrival < 1 (47.6%) 1–2 (26.2%) 2–3 (7.1%) 3–4 (9.5%) 4+ (7.1%)	Perceived barriers and knowledge about cervical cancer, screening, and HPV vaccination	Themes analysed (not further outlined)	Cervical screening KAP: • women had never heard of cervical cancer, • cancer did not occur in their community, • had limited knowledge of HPV. Increased susceptibility to cervical cancer: • multiple sexual partners, • unprotected sexual intercourse, • virus transfers through male partner. Barriers to screening: • construct of prevention not present among women, • shyness, feelings of exposure, potential stigma in seeking a Pap test, • transport to appointments and health system navigation, • language barrier and low trust in providers and interpreters, • high cost (though some women received a Pap test as a priority regardless to keep them healthy), • discomfort with male translators from their community, • women unsure what to ask for due to disparity in health education. Enablers included trust in providers and interpreters and community health workers as health interventionists.	.75
Kim et al. (2017) [58]; HC South Korea; CO North Korea	Explore factors associated with Pap test receipt among North Korean refugee women in South Korea	Purposive sampling; Central Seoul area, Global Together office or other community location; semi-structured interviews	*n* = 8; refugees; 40–60+ yrs.; yrs. since arrival 6	Health seeking behaviours and cultural descriptors (not further outlined)	Inductive content analysis	Cervical screening perspectives; • poor knowledge about cervical cancer and Pap test, • cancer worry regarding fear of abnormal Pap test, • low perceived need for preventive care, absence of symptoms main reason for non-receipt of a Pap test, • families social support, influence of family on decision-making and provider gender were key to Pap test receipt, • should be seen by a women provider regardless whether the test is free or not. Enablers to care: • women providers, • receiving family support, • National health insurance with biennial free Pap test screening. Women lack basic knowledge about cervical cancer and believe it is fatal and not preventable. National and private free cancer screening program available so newly arrived women were more likely to be screened	.90
Lor et al. (2018) [59]; HC USA; CO Bhutan, Burma	Understand factors contributing to Burmese and Bhutanese refugee women’s decisions about cervical cancer screening.	Convenience sampling; King County Washington, private community locations; 8 FGDs (4 Burmese, 4 Bhutanese)	*n* = 58; refugees; 20–50+ yrs.; yrs. since arrival 0–4 (57%) 5–9 (43%)	Factors influencing cervical cancer and screening knowledge, attitudes and experiences	Thematic analysis	Cervical screening decision-making: • shaped by experiences in country of origin, • only sought health care when symptomatic, • unfamiliar with preventive care, • feelings of fear and mistrust about seeking reproductive health care. Barriers to accessing care: • limited English proficiency, • problems with interpreters, • financial and transport concerns, • navigating the US health care system, • embarrassment and stigma related to cervical cancer and screening, • women unfamiliar with reproductive anatomy leading to confusion about tests specific to cervical screening. Enablers to screening: • trusted women providers and interpreters, • positive relationships with doctors and other providers, • receiving health information from family/ friends while retaining confidence in their own health care decisions. Women need culturally tailored health education and a regular source of care in early resettlement	.90
Allen et al. (2019) [60]; HC USA; CO Somalia	Better understand Somali refugee women’s views on cervical cancer screening for themselves and HPV vaccination for their children, particularly barriers and facilitators	Convenience sampling; Minneapolis – St Paul metropolitan area, Somali-focused community organizations/centres; 3 FGDs	*n* = 31; refugees; 20–69 yrs. (mean 36); yrs. since arrival < 10 (19%), > 10 (74%) (mean 13)	Views on cervical cancer screening, HPV vaccination for women and their children, barriers or facilitators affecting uptake	Thematic analysis	Cancer screening perspectives: Participant’s knowledge varied on details of cervical cancer causes and prevention: • untreated infections, • having multiple sex partners, • genetic predisposition, • use of birth control. Enablers to Pap test and HPV vaccination: • web-based education, phone-based intervention, social media, community-based workshops in Somali language Pap test - some believed the test was done in the vagina to detect cancer, early signs of cancer, or infection. Participants had heard about HPV and vaccination but had limited knowledge about HPV. Pap test and HPV vaccine uptake depended on doctor recommendations and receiving husband or family support	.85
Ross Perfetti et al. (2019) [61]; HC USA; CO Iraq	Understand health and attitudes towards preventive care, including cancer screening, and how they relate to cultural and structural mediators of health	Snowball sampling; Philadelphia community clinic; 3 semi-structured FGDs	*n* = 14; refugees; < 30- > 60 yrs.; yrs. since arrival 1–3	Perspectives on wellness, cancer screening and annual physical exams	Thematic analysis	Cancer screening attitudes: • women avoided visiting a health professional until symptoms were intolerable, • embarrassment and shyness with breast and gynaecological exams, • screenings undertaken if explanations were provided. Knowledge was variable. Cancer is caused by dangerous environments: • radiation and chemical exposure, • unhealthy food and food contaminated, • mental health problems, • genetics, • untreated inflammation. Multi-level problems within hospitals and clinics prevent delivery of care: • long wait times, • inadequate evaluations and treatments, • gaps after and between providers and discriminatory practices, • financial barriers and competing priorities - limited time to engage in health activities. Enablers to care: ‘prevention is better than cure:’ demonstrated knowledge about screening. Despite a lack of cancer screenings services in Iraq, women expressed familiarity with screening.	1.0
Babatunde-Sowole et al. (2020) [62]; HC Australia; CO West Africa	Gain insights into attitudes and understandings about preventive healthcare and screening in Australian-West African women in New South Wales, Australia.	Purposive and snowball sampling; New South Wales-African community groups and associations centres; semi-structured interviews	*n* = 22; refugees; 26–62 yrs.; yrs. since arrival 1–15	Healthcare habits prior to and following migration, attitudes to and use of preventive healthcare and screening	Thematic analysis	Cervical screening perspectives: • low health literacy in relation to healthcare practices and disease aetiology, • risk factors related to family history, pollution and being sexually active, • concepts of screening and preventive care unfamiliar • competing priorities such as family commitments took precedence over seeking preventive care • cultural and curative practices of waiting for symptoms of disease before seeking care • uncomfortable with a male healthcare provider • discomfort is a concern due to previous experiences of rape, sexual violence and screening itself. Enablers to care: • increase preventive health awareness within their communities, • self-collection of Pap smears - more convenient and private, • availability of women health providers for SRH, • belief that prayer and race are protective against disease. Affordable healthcare in Australia did not change beliefs and attitudes towards screening/preventive health care – seen as indulgent luxury.	.85
SRH topic: breast screening							
Saadi et al. (2015) [63]; HC USA; CO Somalia, Bosnia, Iraq	Explore Bosnian, Iraqi, and Somali women refugees’ beliefs about preventive care and breast cancer screening	Convenience and snowball sampling; General Hospital Chelsea Healthcare Centre, Massachusetts; semi structured interviews	*n* = 57; refugees; Bosnian 41–75 (mean 54) Somali 27–58 (mean 40) Iraqi 23–55, (mean 41); yrs. since arrival Range < 1 mth to 16 (mean 6)	Knowledge about preventive health care and screening exams for women’s health, knowledge about and barriers to mammograms.	Thematic analysis	Breast screen perspectives and barriers: • fear of pain and diagnosis, modesty, • work and childcare commitments, • varying degrees of medical exposure to doctors in home countries, • home country medical systems focus on acute, not preventive care, • navigating and understanding the host country appointment system, • impact of war on health systems; understanding preventive breast care, SRH did not exist in home countries [Somalia]. • despite awareness of mammography, few Iraqi women had it before resettlement. Enablers to breast screening: • outreach efforts, • appointment reminders, assistance with scheduling and personal contact from HCPs, • perceptions of how medical infrastructure compared with home countries inadequacies, • positive attitude toward HCPs with the increased level of attention and care received, • someone who spoke their language and could explain what was expected, Women across groups indicated willingness to overcome systemic barriers and personal fears of pain or bad news.	1.0
Parajuli, et al. (2019) [64]; HC Australia; CO Bhutan	Explore perceptions and perceived barriers of Bhutanese refugee women accessing and using breast-screening.	Purposive sampling; Melbourne participants homes; in-depth interviews	*n* = 14; refugees; 50–70 yrs.; yrs. since arrival 4–7	Experiences of accessing cervical and breast-screening services	Interpretative Phenomenological Analysis	Breast screening perspectives and barriers: • lack of knowledge about screening importance in detecting problems - though women knew of breast cancer, • lack of motivational factors - doctor had not raised breast screening as important, • problem-triggered health seeking behaviour due to strong cultural factors, • feelings of embarrassment exposing certain body parts, • communication difficulties due to poor literacy and limited English language proficiency, • disliked sharing sensitive health information with their children acting as interpreters, Enablers to care: mammogram undertaken following a doctor’s recommendation	.90

*N/S* not stated, *HC* host country, *CO* country of origin

**Table 2:** Characteristics of quantitative studies and main findings

First Author, (Year); HC; CO	Stated study objectives	Recruitment method; Setting; Data collection method	Sample size; Classification; Age range; Yrs since arrival	Outcome measures	Data analysis	Main results; Conclusions	Quality score
SRH topic: Family planning (FP)							
Morrison (2000) [47]; HC Thailand; CO Cambodia	As outlined in Table 1	Walking through camps women were randomly asked to participate; Khao Phlu refugee camp Thailand maternal and child health centre; survey	*n* = 102; As outlined in Table 1	Contraceptive knowledge, beliefs and practices. Perceptions about FP	N/S	Contraceptive knowledge and use: • 82% of married women wanted to stop or delay childbearing, • 12% reported using a modern method of contraception, • 61% mentioned fear of side effects, • 24% cited lack of information on contraception, • 42% reported discomfort over seeking contraceptives, • 32–48% of women unaware contraceptive methods were available at refugee health centre and none knew about emergency contraception.	.64
Raheel et al. (2012) [65]; HC Pakistan; CO Afghanistan	Measure differences in knowledge and practice of contraception between healthcare subsidised and unsubsidised groups	Systematic random sampling to select households; Karachi settlements of Afghan refugees; questionnaire survey	*n* = 650; refugees; subsidy/no subsidy mean 33%/ 30%; yrs. since arrival subsidy/no subsidy mean 10/13	Knowledge and practice about FP and contraceptive use with and without healthcare subsidies	SPSS Mean/SD Binary logistic regression Adjusted odds ratios 95% CIs	Family planning awareness and use: • 90% in subsidised group aware of FP, compared to 45% in unsubsidised group, • use of contraceptives > 2-fold in subsidised group versus unsubsidised, • access to subsidised care more likely resulted in contraceptive use with advancing age as compared to unsubsidised care. Positive attitude towards FP and higher contraceptive use among Afghan women receiving a healthcare subsidy compared to those not receiving a subsidy despite their conservative background and marginal economic status.	1.0
Kisindja et al. (2017) [66]; HC Congo; CO Congo	Investigate reproductive health and FP knowledge and needs of newly internally displaced women in North Kivu province.	Convenience sampling door to door; two Mugunga displacement camps; verbally administered survey	*n* = 155; internally displaced; 14-45 yrs. (mean 28); yrs. since arrival < 1 yr (34%) < 2 yrs. (95%)	Reproductive health history, contraceptive use, and FP exposures, knowledge and desires	N/S	Contraceptive knowledge and use: • 84% previously received information on contraception, • 35% women knew of at least two contraceptive methods, • 31% reported ever using contraception, • 62% cited lack of interest, 21% lack of knowledge and 12% religious’ opposition for never using contraception Contraceptive knowledge was moderate actual usage was low.	.73
Tanabe et al. 2017 [51], HC Bangladesh Jordan Djibouti Kenya Malaysia Uganda; CO Myanmar, Iraq, Democratic Republic of Congo, Somalia	As outlined in Table 1	Sampling frame – UNHCRs database and registered mobile phone and spatial sampling; Multiple country locations - refugee camps, settlements, urban areas; household survey	*n* = 2733; refugees; 15–49 yrs.; yrs. since arrival N/S	Contraception- awareness, ever use, current use, and unmet need for FP	Descriptive frequencies Binary logistic regression	Contraceptive awareness and use: • 74% reported awareness of at least 1 modern method of contraception, • 48% married women reported ever use of modern contraceptives significantly < unmarried women 16%, • 26% married women reported currently using any modern method to avoid or delay pregnancy, significantly > unmarried women 3%, • 7% of women reported unmet need for contraception, • Married women were over 7 x more likely to report unmet need compared with unmarried women.	1.0
Raben and van den Muijsenbergh (2018) [67]; HC Nether-lands; CO various	Examine the extent Netherlands General Practitioners discuss and prescribe contraceptives to female refugees compared with other female migrants and native Dutch women	Extracted data from General Practice surgery databases; Nigmegen, Rotterdam and Amsterdam, five General Practices; database searches	*n* = 104; refugees; 15–49 yrs.; yrs. since arrival mean 6.5 yrs. (range < 1–34)	Contraceptive method discussed or prescribed with General Practitioner	Two-tailed Pearson chi-squared test, independent samples t-test, one-way ANOVA, binary logistic regression	Contraception access: • 51% General Practitioners reported discussing contraceptives with women refugees, significantly < other migrants, 66% and < native Dutch women, 84%, • in women from Sub Saharan Africa, contraception was significantly less often discussed, 29% compared with refugee women from other regions 68%. Contraceptives were discussed or prescribed significantly < with refugees and other migrant women compared with native Dutch women.	.86
Pierce (2019) [68]; HC Jordan; CO Palestine	Examine regional coverage, source, and method of contraceptives; variation in reproductive health and social experiences by source of contraception; influences on utilisation of reproductive health services	Recruitment method N/S; Jordan- urban area refugee camp households; demographic and health survey	*n* = 10,105; refugees; 15–24 (13%) 25–39 (56%) 40–49 (31%); yrs. since arrival: multi-generational displacement	Modern contraceptive use, FP education at a health facility, contraceptive advice from medical personnel, source of contraception	Descriptive statistics Logistic regression of reproductive health odds ratios for background variables	Contraceptive use and intention: • 14–43% used contraception, 15–55% contraceptive source (govt, pharmacy, private) used, 5–13% modern contraceptive method used, • educational attainment, age, employment, number of living children, and wealth had a significant effect on modern contraception use, • refugee camp existence significantly increases the likelihood of talking about FP at a health facility, • women with large numbers of children > 13 x more likely to utilise UN relief agencies for contraception than those with fewer children. Women living in refugee camps have greater access to FP resources	.91
SRH topic: cervical cancer screening and breast cancer screening							
Barnes et al. (2004) [69]; HC USA; CO Cuba, Bosnia, Vietnam, and others	Explore reproductive health concerns of Bosnian, Cuban, Vietnamese and other refugee women in the US	Recruitment method N/S; Refugee Health Screening Program at local health department; review of medical charts	*n* = 283; refugees; 18–74 yrs. (mean 34); yrs. since arrival N/S	Self-reported medical history, reproductive health problems identified, referrals made, and prescriptions written	Descriptive statistics, z approximation test	Breast and cervical screening practices: • 14% had at least one mammogram, • 86% had never had a mammogram, • 67% of women in the US had at least one mammogram for screening, • rates of mammogram differed between US and refugee women significantly, • 24% of refugee women had a Pap test within the past 3 yrs. compared to US women 79%.	.77
Redwood- Campbell et al. (2008) [70]; HC Canada; CO Albania	Describe reproductive health and mental health-related issues among Kosovar refugees settling in Hamilton, Ontario Canada	Random selection of phone numbers - fieldworkers contacted families; Hamilton, Ontario (not further described); survey questionnaire	*n* = 161; refugees; (18-49 yrs) > 18 yrs. 85 18-49 yrs. 65 > =50 yrs. 19; yrs. since arrival N/S	Ever had a Pap smear, ever heard of a Pap smear, use of contraception, how to access contraception, ever had or heard of a mammogram	Descriptive statistics	Contraceptive use: • 14% reported using some form of contraception Screening awareness and access: • > 50 yrs., 5% of Kosovar women had ever received a mammogram, • 34% of women had ever received a Pap smear, of these 85% had received service in Canada, • Kosovar women reported cervical and breast cancer screening rates in the home country or since arrival were significantly < Canadian rates. Women have little or no history of routine preventive care similar to that which exists in Canada	.50
Lofters et al. (2011) [71]; HC Canada; CO Middle East, North Africa, East Asia, the Pacific, Sub-Saharan African	Determine the independent effects on cervical cancer screening of; sociodemographic factors, health care system, culture and migration for immigrant women in Ontario.	Recruitment method N/S; Ontario’s central metropolitan areas; Data extraction from Landed Immigrant Data System database	*n* = 455,864; refugees; 18–66 yrs.; yrs. since arrival N/S	Women identified as appropriately screened - at least one Pap test in the 3 yr study period	Stratified multi-variate analysis Multi-variate Poisson models stratified SAS for adjusted relative risks	Factors associated with lack of screening: • not being in the 35–49 yr age group, • resident in lowest-income neighbourhoods, • not being in a primary care patient enrolment model, • not having a provider from the same region, • not having a woman provider. For all women, the highest population-attributable risk was seen for not having a woman provider: • 17% for Middle East and North Africa, • 27% for East Asia and the Pacific. Immigrant class was only significant for Sub-Saharan African women and Western European women, with refugees being at > risk of non-screening in these two groups. Women should connect with the health care system soon after arrival and find a regular source of primary care.	1.0
Haworth et al. (2014) [57]; HC USA; CO Bhutan	As outlined in Table 1	Convenience sampling; Burmese community venues and residences Omaha; online survey tool	*n* = 42; As outlined in Table 1	Perceived susceptibility to and severity of disease and perceived barriers and benefits to screening	Descriptive statistics	Cervical cancer and screening practices: • 22% reported ever hearing of a Pap test, • 14% reported ever having Pap test, • 33% perceived susceptibility to cervical cancer, • 71% women who had heard about Pap tests tended to believe more strongly about curability if discovered early compared to 45% of women who never heard about the test A significant lack of knowledge exists in this community regarding cervical cancer and screening practices. Community health workers as health interventionists was well received.	.64
SRH topic: General physical examination							
Odunukan et al. (2015) [72]; HC USA; CO Somalia	Understand Somali women’s comfort with components of physical exam by providers and interpreters of different genders and races	Convenience sample; Mid-west United States, Primary Care Internal Medicine Clinics; pictorial survey	*n* = 50; refugees; 18–90 yrs. (median 46); yrs. since arrival median 11 yrs. (range 0.2–30)	Participant comfort level with body parts being examined by the pictured physician Patient–interpreter gender concordance acceptability	Descriptive statistics Paired ratings of discomfort Bowker’s test of symmetry Simple linear regression	Physical examination: • 98% reported “no problem” to physical examination by a woman provider, • genitalia/pelvic examination (82%), breast examination (81%), and abdominal examination (71%) by male providers was “definitely a problem”, • chest/back examination (29%), extremity examination (28%), and head/neck examination (25%) by male providers was “definitely a problem”. Women preferred a woman provider for conducting examination for the pelvic, breast, and abdominal examinations and preferred woman interpreters to be present.	.77
SRH topic: Female genital cutting							
Banke-Thomas et al. (2019) [73]; HC USA; CO Somalia	Assess factors that influence maternal and reproductive health access across four health care dimensions	Community networks using snowball sampling; Franklin County, Ohio – participants residences; community-based survey	*n* = 427; refugees; 18–19 yrs. 46 20–34 yrs. 215 35–49 yrs. 166; yrs. since arrival < 4 yrs. 139 > 4 yrs. 288	Willingness to seek care, gaining entry to the health system, seeing a primary provider and seeing a specialist	Descriptive statistics Multivariate analyses. Cross tabs bivariate analysis odds ratios 95% CI and *p* values	Factors unique to Somali refugee population: • younger, single women were more willing to seek care vs older, married women. • 81% stated not having insurance was the most frequent reason for postponing public or private care • minors were 2.5 x more willing to seek care than those who arrived in the US as adults Those with insurance were at least: • 2 x more willing to seek care • 3 x more likely to enter the health system • 3 x less likely to have difficulty in seeing a primary provider • odds of Somali women not able to speak English well, being willing to seek care was almost 80% < those who were able to speak English very well • odds of Somali women with female genital cutting being willing to seek care were about 50% < those who were not circumcised	1.0

*N/S* not stated, *HC* host country, *CO* country of origin

### Study locations

Of the 28 studies, 13 were conducted in the USA [48, 53, 55–57, 59–61, 63, 69, 72–74], two each in Australia [62, 64], Jordan [52, 68], Lebanon [49, 50] and Canada [70, 71], one each in Congo [66], Israel [54], Netherlands [67], Pakistan [65], South Korea [65], Thailand [47] and one was a multi country study which included Bangladesh, Jordan, Djibouti, Kenya, Malaysia and Uganda [51].

### Data collection methods

Studies were qualitative (*n* = 16), cross sectional surveys (*n* = 8) and mixed methods; a combination of either focus group discussions (FGDs) or semi structured interviews and surveys (*n* = 4). Of those using qualitative methods, nine involved FGDs [49, 50, 53, 55–57, 59–61], four semi structured interviews [49, 52, 58, 63], three in-depth/key informant interviews [47, 56, 64], three both FGDs and in-depth interviews [48, 51, 54] and one used a novel storytelling method [62]. Of the 12 studies using quantitative methods, nine used face-to-face interview based surveys in respondents’ homes [47, 51, 65, 66, 68, 70, 73] or in a medical clinic [72] and one used an online survey [57]. The remaining studies used population-based data sources (electronic medical and national immigration record databases) [67, 71] and review of medical charts [69].

### Study samples

Twenty-six studies focused on refugees [47–53, 55–65, 67–74], one on asylum seekers [54] and one on internally displaced persons [66]. Five studies included perspectives of HCPs (not further specified) [48, 51, 74] and one each of physicians [54] and nurses [50]. Ethnic groups included Somali /Somali Bantu in seven studies [48, 53, 55, 56, 60, 72, 73], Syrian [49, 50, 52] and Bhutanese [57, 59, 64] in three studies each, and one each in Afghani [65], Albanian [70], Burmese [59], Cambodian [47], Congolese [66], Eritrean [54], Iraqi [61], North Korean [58], Palestinian [68], West African [62] or a combination of these ethnic groups [51, 63, 67, 69, 71]. Participants’ ethnicity was not described in one study [74]. Qualitative data were available from 994 participants (samples ranged between 5 and ~ 65) and quantitative data were available from 469, 984 participants (samples ranged between 42 and 455,684).

### Recruitment methods

Recruitment methods were described in all but one qualitative study [51]. They included convenience sampling with [48, 52–55, 61–63] or without snowball sampling [49, 60] through community or health centres, migrant resource centres or places of worship. Purposive sampling was used in studies of refugee and displaced women and HCPs in various primary health care settings [50, 51, 56, 58, 62]. Several studies engaged community gatekeepers such as community activists, group leaders or women community partners in recruitment of participants [47, 50, 54, 55, 59]. Of eight quantitative studies, four recruited convenience samples [66, 72–74], two obtained data from random samples of households [65] and families [70] and two employed medical record searching [67, 69].

### Quality assessment

The quality of the 28 studies varied considerably with KMET scores ranging between 0.60 and 1.0 for qualitative and 0.50 to 1.0 for quantitative studies. Twenty-two studies reported obtaining ethics approval and six did not. Quality assessment is summarized in Additional files 3 and 4.

### Type of SRH topic examined

All but two studies included at least one of the three main SRH topics: contraception [47–56, 65–68], cervical cancer screening [57, 58, 60, 62, 71, 74] or breast screening [63, 64] with the remaining three examining a combination of these [61, 69, 70]. One examined access to primary care for women who had experienced female genital cutting of different types [73] and one women’s preference for providers of general physical examinations which include breast and pelvic examinations [72].

### Thematic extraction of findings

Table 3 summarises the three main themes and 10 subthemes identified in this review.

**Table 3:** Summary of main themes

Main themes	Sub themes (Country setting – High-income country-HIC/ Low-middle-income country-LMIC)
Interpersonal and patient encounter factors - patient interactions with health care systems and HCPs	Knowledge, awareness and use of preventive SRH care (HIC and LMIC)
	Perceived need for preventive SRH care (HIC and LMIC)
	Language and communication (HIC and LMIC)
Health care system factors – health system factors and their impact on outcomes	Health care provider discrimination and lack of quality health resources (HIC and LMIC)
	Financial barriers and unmet need (LMIC)
	Health care provider characteristics (HIC and LMIC)
	Health system navigation (HIC and LMIC)
Sociocultural factors and the refugee experience - the influence on outcomes of refugee and resettlement experiences.	Family influence (HIC and LMIC)
	Religious factors (LMIC)
	Cultural attitudes (HIC and LMIC)

#### Interpersonal and patient encounter factors - patient interactions with health care systems and HCPs

##### Knowledge, awareness and use of preventive SRH care

*Contraception* Almost all studies on contraception reported some lack of knowledge, awareness and barriers to uptake among women [47–49, 51–54, 56, 66, 68]. Negative beliefs or misperceptions about side effects of contraceptive methods were evident in three high quality qualitative studies of Somali and Syrian women residing in the US and Lebanon [49, 53, 56]. Inaccurate beliefs about contraception included; consequent inability to conceive after discontinuing contraception and decreased sex drive [53], and fear that modern contraceptives cause infertility [56], menstrual irregularities and mood disorders [49]. Six studies conducted in Jordan, Democratic Republic of Congo, Thailand and other low-income countries assessed whether women had ever used contraceptives [47, 51, 66, 68], their awareness of methods of contraception [51], and information received about contraception [66]. A high quality qualitative study of Syrian women in Jordan found they had high awareness of modern contraceptive methods but limited access to contraceptive counselling and services [52]. High awareness and low use of contraception was more evident in low compared with high-income countries due mainly to overburdened health services, cultural pressures regarding fertility, poorly trained service providers, health service disrupted by conflict, distance to service delivery points, cost of transport, religious opposition, language barriers with providers, and provider biases [51, 52, 66]. In refugee camp settings contraceptive knowledge was moderate in Afghan refugee women in Pakistan, Rohingya refugee women in Bangladesh [51, 65] to high in internally displaced people in the Democratic Republic of the Congo [65, 66] while actual usage was low in Cambodian refugees in the Khao Phlu Camp in Thailand [47, 66, 70]. Where the value and knowledge of contraception was moderate to high women reported approval of their friends and spouses. Women having discussions with their husbands about the number of children they should have supported contraceptive use [65]. One study reported that women themselves made the decision to use contraception [66]. Conversely, barriers for contraceptive use included the husband making decisions about the wife’s access to birth control, fear of side effects, lack of information, having a current illness, expense, being too old, being unable to obtain them and husband’s refusal to permit contraceptive use [47].

*Cervical and breast cancer screening* Knowledge, awareness and/or uptake of cancer screening was reported in nine studies conducted in high-income countries in the US [59–61, 69], Australia [62, 64], Canada [70] and South Korea [58]. Women’s understanding of causes of disease and disease prevention was reported to be limited in most studies [57–62, 64, 69]. While some participants resettling in the US and Australia had heard of cervical cancer in four studies of Somali and Iraqi women [56, 60–62], studies of other ethnic groups had variable and at times inaccurate knowledge of cancer aetiology [59–62], cancer prevention [57, 58, 61, 62] and the purpose of screening [64, 69]. One study in the US reported Iraqi women would undergo screening only if provided with explanations of the exam and its necessity [61] which corroborates the importance women placed on doctors’ screening recommendations [49, 64]. A study of HCPs’ views in the US reported that women from various ethnicities were unfamiliar with tests to identify disease at its early stages [74].

Less knowledge and awareness of HPV and HPV vaccination compared with cervical screening was evident in two US studies of Somali and Bhutanese women [57, 60] with varying understanding of whether the virus causes cancer [60] (HPV vaccination has been offered in the US since 2006). Cross sectional surveys measuring rates of breast and cervical screening among refugee and displaced women found lower screening rates when compared with the host country populations of the US and Canada [57, 69, 70]. Barriers to screening identified in Somali and Bhutanese refugee women and refugee women from other ethnic groups were limited or no knowledge regarding cervical cancer and its prevention [57, 60].

##### Perceived need for preventive SRH care

Unfamiliarity with preventive care and low perceived need for it was apparent in seven studies of Somali, Iraqi, Bhutanese and North Korean women conducted in the US [56, 57, 59, 61], Australia [62], Canada [70] and South Korea [58]. The belief that only symptomatic women need to undergo screening was common in eight studies [56–59, 62–64, 69], with absence of symptoms being one reason for not having cervical [58] or breast screening [64]. This resulted in women not seeking preventive care [62, 64] or delaying seeking care until a problem became unbearable [61]. One high quality study of Bosnian, Iraqi and Somali women found doctors in some countries of origin focused only on acute, not preventive care [63]. A study of US HCPs' views found that refugee women often sought care only when symptomatic [74].

##### Language and communication

Access to care related to communication was reported in four studies on contraception [48, 51, 54, 56], three on cervical and breast cancer screening [57, 59, 64] and one on female genital cutting [73]. Limited English language proficiency [57, 59, 64, 73] and poor literacy impacted on access to care [64, 73] and were challenging for HCPs and Somali and Eritrean women alike [48, 54, 74]. In two studies the real communication barrier was reported to be that telephone or in person interpreters did not understand the concepts being discussed [48, 59]. Other studies reported few, if any, translation services available in any healthcare facilities [54, 59], HCPs being unaware of language requirements [48] and women feeling rushed and unable to see or hear telephone interpreters [59]. Communicating with interpreters and providers during reproductive health consultations was reported to lead to embarrassment, shyness or stigma for some women [47, 48, 59, 61, 63, 64].

#### Health care system factors – health system factors and their impact on outcomes

##### Health care provider discrimination and lack of quality health resources

HCP discrimination described as poor communication and perceived lack of care [54], judgemental approaches and disrespectful behaviour in the provision of contraception impacted on refugee women in low income country settings in access to care [50]. A lack of affordable and acceptable methods of contraception offered to women in Jordan and other refugee camp settings, [51, 52] was also identified as a barrier to contraceptive use in studies of Syrian and Eritrean women in low-income country settings [50–52, 54]. HCPs in Lebanon reported their own discrimination in the form of negative attitudes towards Syrian women; one study suggesting women were irresponsible, unreliable and ignorant when seeking care [50]. Other studies in low-income countries indicated poor or second-class treatment occurred due to poor communication, inadequate length of time required address women’s needs, providers own attitudes and fears towards asylum seekers in Israel [54] and gaps in their own skills and knowledge in providing care [51].

Studies of HCPs’ views conducted in low-income countries of Lebanon, Israel and others found limited acceptable contraceptive methods impacted on accessing care [50, 51, 54]. An associated lack of quality resources and perceptions of low-quality care such as provision of incorrect information, incorrect prescriptions, poor hygiene in SRH care were cited in high- and low-income settings [51, 54, 55]. Women’s negative clinical encounters with HCPs in low-income country settings coupled with fear of maltreatment when seeking contraception, unwillingness to have contraception prescribed and time constraints were reported experiences of discrimination [47, 50, 54]. Discrimination appears to have continued following resettlement. One Dutch study reported fewer discussions about contraception between General Practitioners and refugee and displaced women compared with other migrants or host country populations [67]. Another US study reported poor recognition of women’s experience of physical pain by their HCP [61]. Fear and mistrust in seeking care as a result of trauma, sexual violence and bad experiences of screening and diagnosis were also evident among refugee women of various ethnicities in studies conducted in the US and Australia [59, 62, 63].

##### Financial barriers and unmet need

Cost was cited as the main barrier to preventive care by many women [49, 50, 54, 59] and HCPs [54] particularly in low-income settings where access to free healthcare was not universal. Low awareness of the availability of free contraceptive services was cited in two studies in Lebanon and Cambodia [47, 49]. Afghani and Palestinian refugee women in informal settlements and refugee camps in neighbouring countries were between two and 13 times more likely to use contraceptives if costs were subsidised [65, 68]. A South Korean study of women refugees from North Korea found cost deterred women from cervical screening [58]. In one high quality study, lack of health insurance had a strong association with women’s access to maternal and reproductive health care across the continuum of primary care services including family planning and was the most common reason for postponing a visit to primary care [73].

##### Health care provider characteristics

In several studies women from different ethnic groups expressed concern about receiving care from male HCPs [58, 59, 62, 72] with some choosing to forgo care rather than discuss reproductive health topics or undertake cervical screening [48, 71]. Odunukan and others found Somali women preferred a woman provider for physical examinations [72]. HCPs showed insight into their own limitations with regard to caring for refugee and displaced women. Despite women being receptive to screening information in early resettlement, HCPs reported that lack of knowledge, concerns about the timing of information delivery and their own discomfort were barriers to discussing screening [74].

##### Health system navigation

The complexity of healthcare systems impacted on access in seven studies, though the nature of the difficulties varied according to low- or high-income country setting [47, 51, 54, 57, 59, 63, 69]. In low-income settings distance to service point, lack of transport and the fragmented nature of service provision [51, 54] were identified as barriers to contraceptive care even when clinic opening times/ locations and contraceptives were available [54]. Navigating and understanding the health system and making appointments were barriers reported in high-income host countries [59, 63, 69].

#### Sociocultural factors and the refugee experience - the influence on outcomes of refugee and resettlement experiences

##### Family influence

Ten studies in both high- and low-income country settings suggested family influence affected women’s utilisation of contraception, breast and cervical screening [47–53, 55, 58, 60]. Husband’s resistance to birth control and having the final decision about contraceptive use [49, 51, 53] impacted on access. Acceptance by women about the dominant role of the male partner in the decision-making [50, 52], family’s interference [49] as well as deferral to HCPs decision making [48] also impacted on contraceptive use across high and low-income countries. HCPs in Thailand reported that Cambodian women were considered candidates for contraception only after approval from their husbands [47] despite women wanting to stop or delay childbearing [47]. Conversely, a Jordanian study of displaced Palestinian women serviced by the United Nations Relief and Works Agency found they had greater access to health-related resources such as contraception than the host population [68].

##### Religious factors

Religious opposition to contraception was apparent in high and low-middle income country settings [48, 51, 53, 56, 66]. It was taboo for a woman to state she did not want more children [53], nor to use modern birth control as it was forbidden by religion [48]. Religious teachings actively discouraged modern contraceptive use in five of the six population groups included in one multi-country study [51]. The importance of marriage and fidelity for Cambodian women in Thailand created a barrier to using contraception [47]. However, in other ethnic groups such as internally displaced Congolese women, religious opposition to contraception was low [66]. Amongst Somali women resettling in the US, contraception was more acceptable when framed as temporary assistance for birth spacing as this agreed with the tenets their religion [55]. SRH services differ in each resettlement country. This may provide insight on why the barriers to contraception varied across ethnicity and host country. HCPs in the US reported that despite there being strong opposition to contraception in Somali culture and religion, they asked for and used contraception upon resettlement [48].

##### Cultural attitudes

Traditional cultural attitudes towards fertility influenced modern contraceptive use following displacement [51, 52, 56, 73]. In one qualitative study of Syrian women in Jordan, pressure to marry and begin childbearing early was the main barrier for some (young and unmarried) women but not others [52]. In Somali culture, one US study reported that contraception is not discussed with young unmarried women and girls as pre-marital sex is stigmatized and disapproved of [56]; a finding supported by another study among unmarried Iraqi, Burmese, Rohingya, and Somali women in refugee resettlement camps in several low-income countries [51].

Engagement with HCPs was also reported to influence access to contraception. In a group of Somali bantu women in the US considerable cultural deference given to authority figures and an accompanying lack of self-advocacy in interactions with HCPs influenced access [48]. Tanabe and colleagues [51] reported that emergency contraception was offered to survivors of sexual assault by HCPs working in a gender-based violence program in refugee resettlement camps in low-income countries. However, they reportedly disapproved of making emergency contraception available for non-sexual assault cases, citing it could promote promiscuity [51]. For Somali women resettled in the US, being circumcised was found to impede entry into the health system and access to primary HCPs [73].

Positive relationships with and positive attitudes towards doctors and other HCPs were reported to improve access to care by Burmese and Bhutanese arrivals in the US [59, 63]. A key theme for women from these ethnic groups was the preference for women providers due to the cultural considerations of modesty and privacy and the association with honour and virtue. Other studies in the US and Jordan found the presence of trusted woman providers and interpreters improved the cultural acceptability of services and increased uptake of contraception and cervical screening [52, 59, 71, 74]. Intervention strategies to promote cervical screening and HPV vaccination were identified in three studies. They were language and culture specific web, telephone and community-based programs [60], free biennial screenings [58] and screening self-collection which is a particularly effective intervention for refugee and displaced women for reasons outlined above [62]. Congolese women resettled in the US and Syrian women resettled in Lebanon reported more equal relationships and increased empowerment regarding women’s changing roles in decision making about contraceptive use following resettlement [50, 55]. Desire for educational achievement and the absence of extended family were identified as factors facilitating contraceptive use in one US study of Somali women [56]. Support from husbands in health decisions, encouragement from partners and other family members were reported to impact positively on women’s decision to have cervical screening in the US and Canada [58, 60]. Interpreters conveying educational messages about cervical screening and HPV vaccination were reported to increase take-up of screening [57, 60]. Furthermore, interpreters in the US with some medical knowledge who were members of the Somali refugee community and acted as ‘refugee mentors’ improved access to care [73].

## Discussion

To our knowledge this is the first systematic review of the evidence that explicitly addresses access to preventive SRH care for refugee, asylum seeker and internally displaced women. Most research has focused on identifying barriers, much less on enablers of access to care. There was consistent evidence about three factors impacting on access: patient experiences of clinical encounters, the host countries’ healthcare systems and the sociocultural context of a refugees’ journey.

There are few investigations of HCPs’ perspectives and their experiences of providing SRH care for refugee and displaced women. This limits the opportunity to ascertain consistencies and inconsistencies between refugee and displaced women and HCPs’ views. Most investigations were of refugees with only one each of internally displaced people and asylum seekers limiting the opportunity to compare the differences between these groups.

### Strengths and limitations of the systematic review

A comprehensive search of five databases was conducted using a published protocol. It was supplemented by citation searches, so all relevant studies were likely to have been identified. Study selection bias was minimised by having pre-set inclusion criteria and having three authors undertake full text screening independently, with differences in opinion resolved in discussions among all authors. Two authors independently assessed the quality of the included studies using a well-established quality assessment tool [45].

The varied study designs, and their use of qualitative, quantitative and mixed methods precluded meta-analysis of findings. However, the inclusion of methodologically diverse studies and the narrative synthesis enabled a comprehensive understanding of SRH care access for refugee and displaced women. The foundation of the qualitative evidence was complementary to quantitative results, presenting a complete picture of women’s experiences. For instance, quantitative studies estimated prevalence of contraceptive knowledge, contraceptive information provision, and prevalence of use of contraception amongst refugee and displaced women. Whereas, qualitative studies elucidated the reasons why women’s uptake of contraception was low in some settings. Qualitative studies also described women’s views on their decision to use contraception and perceptions of service availability, accessibility, and quality.

Similarly, quantitative studies established rates of breast and cervical screening as well as cancer knowledge and awareness among refugee and displaced women. Whereas qualitative studies provided details on the overlapping complexities and context of why women were aware of breast and cervical screening but had not accessed the services, despite its availability in high income countries.

A strength of the narrative synthesis is that it enabled overlaying of themes across studies. It also allowed the comparison of findings and relationships between studies. When interconnected points of similarity are identified and shaped in a narrative synthesis, findings are likely generalisable and can be used to inform policy and practice [75].

The exclusion of non-English language articles may have meant that relevant publications in other languages were not included. We therefore acknowledge that the included articles may not reflect all cultural perspectives. Furthermore, it is possible that the searches did not identify studies where refugees, asylum seekers or internally displaced people were referred to as ‘migrants’ or ‘immigrants’. The selected studies focused mainly on women with refugee status already determined in the destination country. There may be additional barriers for internally displaced people and asylum seekers that are not described fully in this review due to the limited number of studies of women in these groups. The limited number of internally displaced people and asylum seeker studies also makes it difficult to draw subgroup comparisons.

### Strengths and limitations of the included studies

Overall, the quality of the studies was high with none judged as poor quality. Study designs and methodologies were appropriate for the studies’ aims and closely aligned with their outcome measures. A strength of both qualitative and quantitative studies was that most data came from primary sources - the women themselves, through a variety of methods. The definition of ‘refugee’ was consistent across studies which suggests the results reflect the experiences of refugee and displaced women.

Of the qualitative studies, few reported on the quality assessment criteria of “reflexivity”. Reflexivity is the acknowledgement of cultural differences and asymmetry in the researcher - participant relationship and how these may affect the study method used, participant recruitment, and study findings and conclusions [76].

### Methodological quality

Many studies used convenience sampling strategies. This may have resulted in an overrepresentation of women already seeking care, or a bias toward those with greater ability to navigate the health system. The studies would more accurately represent the factors impacting on access to care had recruitment gone beyond and included community members yet to engage with health and community services. Qualitative studies used either focus groups or interviews; methods considered the most appropriate for eliciting views on sensitive topics in vulnerable populations [77, 78]. Studies used face to face bilingual researchers or interpreters, a recognised method for overcoming challenges of participants low literacy and/or low health literacy [79].

Quantitative studies were strengthened by using validated survey instruments translated into participants’ language. However, some instruments had not been cross-culturally validated and back translated. Most surveys were administered individually and verbally, modes of delivery considered the least burdensome for those with low literacy [80].

### Overview of findings

There are many barriers to SRH care in both high and low- and middle-income country settings, including women’s low perceived need for preventive care, cultural attitudes and beliefs about family planning, cervical and breast cancer screening and HPV vaccination, stigma and shame surrounding women’s access to and use of SRH care services, discriminatory practices, lack of women HCPs, culturally competent care, and language barriers. However, our review indicates that many barriers are exacerbated by the refugee context and that additional barriers to SRH exist in LMIC settings. Deficiencies in infrastructure and transport, costs of transport to access services, and fragmentation of the health care system, especially in refugee camp settings and in poorly resourced resettlement countries, limit women’s access to SRH care and support. Male partners’ influences in decision-making about women’s SRH can limit access to contraceptive counselling and services. Health provider biases in providing contraception, poor awareness of SRH services, a lack of privacy and confidentiality, and respectful and woman-friendly SRH services were other barriers in many low- and middle-income resettlement settings.

Conversely, enablers to SRH care were mainly reported in high income countries. Resettlement in high income settings found women’s agency and self-determination are highly valued and are reflected in principles of service provision including for women who are refugees when accessing SRH. This was probably reflected in women reporting greater ownership of their SRH care, [48], increased equality in their personal relationships, [55] and more positive relationships with their HCPs [59, 63]. 

#### Patient experiences of clinical encounters

The breadth of the studies reviewed was reflected in the seventeen different cultural and ethnic backgrounds identified conducted across sixteen high- and low-income countries. Despite this diversity, similar patterns of access to SRH care were reported. One consistent finding was that unfamiliarity with preventive SRH care and low health literacy impacted on accessing care. Key components of health literacy were identified throughout the review; difficulties navigating the health system, lack of knowledge of disease aetiology, poor health care practices, limited sense of self-advocacy and the challenges of taking ownership of one’s health. These factors were exacerbated by women’s lack of autonomy and this quantitative study found both physical and emotional domestic violence had a significant impact on refugee women receiving contraception [68].

Communication barriers were consistently highlighted. Irrespective of women’s ethnic background or host country language barriers coupled with poor cultural competence in service provision impacted on access. Communication barriers are known to create difficulties negotiating health systems [81] and decrease the quality of healthcare once refugee and displaced women are engaged in the system [82, 83]. The lack of and poor quality of interpreters as well as interpreters’ lack of health/SRH knowledge were also highlighted [48]. When discussing SRH, engaging qualified medical interpreters is preferred by refugee and displaced women [84] and has been shown to enhance the delivery of SRH care to women with limited English proficiency [85].

#### Host countries’ health care system

Women consistently reported experiencing discrimination and disrespectful care though this was more evident in low-income countries informal resettlements arrangements [50, 51, 54, 61]. Cultural competence is the ability to participate ethically and effectively in personal and professional intercultural settings. It requires being aware of one’s own cultural values and respectful of others values, beliefs, traditions and customs [86]. Culturally competent practice impacts on health care experiences but is challenging for many HCPs [7, 87]. Poor cultural awareness results in incorrect assumptions about refugee health needs which in turn results in decreased quality of health care and low health service utilisation [88], which was highlighted in both high- and low-income countries in this review.

Gender discordance is the discrepancy between the gender of the patient and the HCP [89]. Patient - provider gender discordance was a consistent barrier across SRH topics, ethnic groups and host country settings. Being seen by a woman provider was a key influencing factor in accessing SRH care [50, 58, 64]. The review highlighted that women were more likely to seek SRH care and be more comfortable expressing their needs with women HCPs and interpreters.

#### Sociocultural context of a refugees’ journey

Many sociocultural barriers also exist for women from migrant backgrounds who are not refugees [90]. However, refugee and displaced women may experience violence, trauma in their countries of origin and prolonged and dangerous in transit journeys prior to arrival in a resettlement country. Consequently, there are additional and more acute sociocultural barriers to accessing SRH for this group upon arrival. These barriers include family, cultural and religious influences as well as knowledge, awareness and use of SRH care [9, 90].

Specific barriers to care for vulnerable subgroups of refugee and displaced women were identified. Younger unmarried women [50] and women who had experienced female genital cutting [73] faced additional layers of disadvantage in accessing SRH care. In this review the patterns of access of those sub-groups were difficult to establish given the limited number of related studies.

There was uniformity of findings relating to positive influences on access to SRH care across ethnic groups and healthcare contexts. Interpersonal factors included establishing trust, confidence and communication between refugee and displaced women and HCPs, HCPs training in cultural competency, provision of quality medical interpreters and patient-provider gender concordance. This suggests building meaningful relational connections, acknowledging refugees’ journey, establishing rapport, taking the time needed to communicate and access to women providers are important in the provision of care to refugee and displaced women.

### Implications for clinical practice and future research

#### Clinical practice

Key solutions to addressing access to care lie in strengthening SRH education for refugee and displaced women, including offering culturally sensitive information about the importance of preventive SRH care and how the health system functions in the host country. Such initiatives are known to lead to increased utilisation of services [91] and better SRH outcomes [74, 92].

Interventions to improve health literacy might include offering preventive SRH education in appropriate languages, targeting reading levels and design of printed material and using the Teach-Back method in face to face education [93], supporting health system navigation, and promoting self-efficacy and self-advocacy skills.

#### Policy

Ongoing HCPs professional development and education encompassing culturally competency and awareness of SRH needs leads to better health care provision of ethnically diverse groups [94]. Cultural competence training has been developed to improve access to care [95], and has resulted in better quality care [96, 97] and reduced discrimination in settings with diverse ethnic groups [98]. Ensuring adequate refugee-specific health services and well-trained culturally sensitive HCPs, such as specialist General Practitioners, refugee health nurses, and bicultural healthcare workers may improve SRH care for refugee and displaced women. Health policy aimed at providing bilingual community support workers and medical interpreters as advocates with knowledge and sensitivity to address SRH topics would benefit women during resettlement. Providing an understanding of the complex SRH care needs of refugee and displaced women in undergraduate and professional development programs would further facilitate access. Ensuring specific health literacy education modules for primary HCPs as a component of their continuing professional development should be a priority. Such initiatives should be a priority in high-income resettlement countries where standards and guidelines can be set and adapted appropriately to low-income country settings with less resources.

Addressing gender discordance by employing more women General Practitioners, increasing the scope of practice for refugee health nurses, and giving women the option of choosing the gender of practitioners may facilitate care. This, together with providing adequate human resourcing allowing longer consultation times to accommodate cultural needs, to listen and develop women’s trust and confidence and confirm screening and contraception information is understood could enhance access to care.

#### Future research

HCPs knowledge and behaviour toward refugee and displaced women significantly impact women’s access to SRH. More research is needed to understand HCPs educational and cultural competency needs. Furthermore, identifying HCPs understanding of health literacy principles to support them in developing strategies towards facilitating women’s health literacy is needed. Identifying additional positive influences on access to care for refugee and displaced women, particularly in the early resettlement period when women feel more empowered to make their own decisions regarding SRH care, should be a priority. Further research in the area of access to preventive SRH care for women who have experienced female genital cutting is also warranted.

## Conclusion

The findings of this review show that to improve access to culturally sensitive and patient-centred care SRH care in primary healthcare settings, interpersonal factors including knowledge, awareness perceived need for and use of preventive SRH care; language and communication barriers, health system factors including HCP discrimination, lack of quality health resources; financial barriers and unmet need; HCP characteristics; health system navigation and sociocultural factors including family influence; religious and cultural factors need to be addressed. The findings can inform practice, public health policy, and health professional education to ensure refugee and displaced women have access to quality preventive SRH care in primary care settings, particularly in low-incomes countries where most refugees seek resettlement.

## Supplementary Information

**Additional file 1.** Selection criteria.

**Additional file 2.** Database search strategy.

**Additional file 3.** Quality assessment of quantitative studies and quantitative component of mixed methods.

**Additional file 4.** Quality assessment of qualitative studies and quality component of mixed methods.

## Data Availability

The datasets used and/or analysed during the current study are available from the corresponding author on reasonable request.

## Abbreviations

CO: Country of origin
FP: Family Planning
FGD: Focus Group Discussion
HC: Host country
HCP: Health Care Provider
HPV: Human Papilloma Virus
OCP: Oral contraceptive pill
SRH: Sexual and Reproductive Health
US: United States of America
