# Supplementary material for: Access to preventive sexual and reproductive health care for women from refugee-like backgrounds: a systematic review
Source: BMC Public Health. 2022 Feb 27;22:403. doi: 10.1186/s12889-022-12576-4 (PMC8882295; doi:10.1186/s12889-022-12576-4)
Supplement: Supplementary file 1 — Additional file 1. Selection criteria. [file 12889_2022_12576_MOESM1_ESM.docx]

**Additional file 1 Selection criteria**

**Study type**

| Include | Exclude |
| --- | --- |
| Primary qualitative, quantitative and mixed methods research studies  Interviews/focus groups  Peer reviewed | Opinion pieces/Commentary  Book Reviews/Literature reviews |

**Health care users**

| Include | Exclude |
| --- | --- |
| Refugees  Asylum seekers  Refugee like backgrounds  Undocumented migrants  Illegal immigrants  Migrants (with refugee group identified)  Immigrants (with refugee group identified)  NB Studies that contained a mixture of eligible and ineligible participants were only included if data for eligible participants could be isolated for synthesis | Affluent migrants such as  Tourist  Expatriates  Economic migrants |

**Health care professionals**

| Include | Exclude |
| --- | --- |
| Primary care Nurses  Nurse practitioners  General Practitioners  Sexual health nurses  Refugee health nurses  Health visitors | Specialists  Allied health professionals  Midwives  Pharmacists  Dentists |

**Settings of practice**

| Include | Exclude |
| --- | --- |
| Primary Health care setting including  General practices  Sexual health clinics  Refugee health clinics  Community Health centres/ clinic | Asylum seeker detention centres  Acute care settings – hospitals  Specialist centres/ appointments  Travel medicine clinics |

**Outcome**

| Include | Exclude |
| --- | --- |
| Studies concerned with the participants’ and health providers views on access, utilisation, barriers or enablers, experiences, perceptions or attitudes to sexual and reproductive health care services in the international setting | Maternity and obstetric care  HIV / STI prevention  Studies concerned with men |
