# Supplementary material for: Access to preventive sexual and reproductive health care for women from refugee-like backgrounds: a systematic review
Source: BMC Public Health. 2022 Feb 27;22:403. doi: 10.1186/s12889-022-12576-4 (PMC8882295; doi:10.1186/s12889-022-12576-4)
Supplement: Supplementary file 2 — Additional file 2. Database search strategy. [file 12889_2022_12576_MOESM2_ESM.docx]

**Additional file 2: Database search strategy**

| MEDLINE | EMBASE | CINAHL | PsychINFO | GLOBAL HEALTH |
| --- | --- | --- | --- | --- |
| 1. Refugees/  2. Undocumented immigrant/  3. Immigrant/  4."Transients and Migrants"/  5. asylum adj2 seek*  6. person*1 adj2 displace* 7. migrant*1 adj3 undocument*  4. illegal*1 adj4 immigrant*  5. illegal*1 adj2 migrant*  6. refugee* adj3 background*  7. humanitarian adj1 entrant*  8. culturally and linguistically diverse  9. ((ethnic* or racial) adj2 (group* or minority))  10. (immigrant* or migrant*).mp | 1. Refugee/  2. Undocumented immigrant/  3. Immigrant/  4. asylum adj2 seek*  5. person adj3 displace  6. migrant adj3 undocument*  7. illegal*1 adj4 immigrant*  8. illegal*1 adj2 migrant*  9. refugee adj3 background  10. humanitarian adj1 entrant*  11. culturally and linguistically diverse  12. ethnic* or racial  13. group* or minority  14. (immigrant or migrant).mp. | 1. "Refugees"  2. "Immigrants, Illegal"  3. "Transients and Migrants"  4. "Cultural Diversity"  5. "Ethnic Groups"  6. "Minority Groups"  7. "Immigrants"  8. (asylum N1 seek*)  9. (person*1 N1 displace*) 10. (person*1 N1 displace*) 11. (migrant* N2 undocument*)  12. (illegal N2 immigrant)  13. (refugee* N1 background*)  14. (illegal* N1 migrant*)  15. (humanitarian N1 entrant*)  16. (culturally and linguistically diverse)  17. ((ethnic* or racial) N1 (group* or minority)) | 1. Refugees/  2. Asylum Seeking/  3. "Racial and Ethnic Groups"/ or Minority Groups/  4. (undocument* adj2 immigrant*)  5. (person*1 adj3 displace*) 6. (migrant*1 adj3 undocument*)  7. (illegal adj4 immigrant*)  8. (illegal adj2 migrant*)  9. (refugee adj1 background*)  10. (humanitarian adj1 entrant*)  11. (culturally and linguistically diverse)  12. ((ethnic* or racial) adj1 (group* or minority))  13.(immigrant* or migrant*).mp. | 1. Refugee/  2. (ethnic groups or minorities).sh.  3. immigrants/  4. migrants/(asylum adj1 seek*)  5. (undocument* adj2 immigrant*)  6. (person*1 adj1 displace*) OR (migrant*1 adj3 undocument*)  7. (illegal*1 adj3 immigrant*)  8. (illegal*1 adj1 migrant*)  9. (refugee adj1 background*)  10. (humanitarian adj1 entrant*)  11. (culturally and linguistically diverse)  12. ((ethnic* or racial) adj1 (group* or minority))  13. (immigrant* or migrant*).mp. |
| 11. 1 or 2 or 3 or 4 or 5 or 6 or 7 or 8 or 9 or 10 | 15. 1 or 2 or 3 or 4 or 5 or 6 or 7 or 8 or 9 or 10 or 11 or 12 or 13 or 14 | 18. 1 or 2 or 3 or 4 or 5 or 6 or 7 or 8 or 9 or 10 or 11 or 12 or 13 or 14 or 15 or 16 or 17 | 14. 1 or 2 or 3 or 4 or 5 or 6 or 7 or 8 or 9 or 10 or 11 or 12 or 13 | 14. 1 or 2 or 3 or 4 or 5 or 6 or 7 or 8 or 9 or 10 or 11 or 12 or 13 |
| 12. Health Services Accessibility/ or "Patient Acceptance of Health Care"/ 13. ((access* or avail* or barrier* or need* or facilitat* or enabler* or strength* or disparit* or experience* or knowledge* or litera*) adj3 (health or healthcare or health care or care or service* or program*))  14. ("use" or us?age or utili?at* or utili?e) adj3 (health or health care or healthcare or health service* or health resource* or patient* accept*)).mp | 16. health care utilization/  17. health care access/  18. ("use" or us?age or utili?at* or utili?e) adj3 (health or health care or healthcare or health service* or health resource* or patient accept*)  19. (access* or delivery* or availa* or barrier* or facilitat* or strength* or disparit* or need* or experience* or knowledge or litera*) adj3 (health or healthcare or health care or care or service* or program*).mp. | 19. "Healthcare Disparities"  20. "Health Care Delivery, Integrated"  21. "Health Services Accessibility"  22. (("use" or us?age or utili?at* or utili?e or delivery) N2 (health or health care or healthcare or health service* or health resource* or patient accept*))  23. ((access* or availa* or barrier* or facilitat* or strength* or disparit* or need* or experience* knowledge* or litera*) N2 (health or healthcare or health care or care or service* or program*)) | 15. Health Care Services/ or Health Care Utilization/  16.Health Care Delivery/ or Health Care Access/  17. (("use" or us?age or utili?at* or utili?e) adj3 (health or health care or healthcare or health service* or health resource* or patient accept*))  18. ((access* or delivery* or availa* or barrier* or facilitat* or strength* or disparit* or need* or experience* or knowledge* or litera*) adj3 (health or healthcare or health care or care or service* or program*)).mp | 14. health care utilization.sh.  15. access.sh.  16. (("use" or us?age or utili?at* or utili?e) adj3 (health or health care or healthcare or health service* or health resource* or patient accept*))  17. ((access* or delivery* or availa* or barrier* or facilitat* or strength* or disparit* or need* or experience* or knowledge* or litera*) adj3 (health or healthcare or health care or care or service* or program*)).mp. |
| 15. 12 or 13 or 14 | 20. 16 or 17 or 18 or 19 | 24. 19 or 20 or 21 0r 22 or 23 | 19. 15 or 16 or 17 or 18 | 18. 14 or 15 or 16 or 17 |
| 16. Women's Health/  17. Women's Health Services/  18. Reproductive Health/  19. Sexual Health/  20. (wom?n* adj3 (health or service*))  21. (health adj3 (wom?n* or sexual or reproductive)).mp. | 21. Womens health/  22. reproductive health/  23. sexual health/  24. (wom?n* adj3 (health or service*))  25. (health* adj1 (wom?n* or sexual or reproductive)).mp. | 25. "Women's Health"  26. "Reproductive Health"  27. "Sexual Health"  28. (wom?n* N3 (health or service*))  29. (health N2 (wom?n* or sexual or reproductive)) | 20. Reproductive Health/  21. Sexual Health/  22. (wom?n* adj3 (health or service*))  23. (health adj3 (wom?n* or sexual or reproductive)).mp. | 19. Women's health.sh.  20. reproductive health/  21. sexual health/  22. (health* adj1 (wom?n* or sexual or reproductive))  23. (wom?n* adj3 (health or service*)).mp. |
| 22. 16 or 17 or 18 or 19 or 20 or 21 | 26. 21 or 22 or 23 or 24 | 30. 25 or 26 or 27 or 28 or 29 | 24. 20 or 21 or 22 or 23 | 24. 19 or 20 or 21 or 22 or 23 |
| 23. 11 and 15 and 22 | 27. 15 and 20 and 26 | 31. 18 and 24 and 30 | 25. 14 and 19 and 24 | 25. 14 and 18 and 24 |
| **1151** | **917** | **700** | **320** | **995** |

| **Total from database searches** | **4083** |
| --- | --- |
