# Supplementary material for: Access to preventive sexual and reproductive health care for women from refugee-like backgrounds: a systematic review
Source: BMC Public Health. 2022 Feb 27;22:403. doi: 10.1186/s12889-022-12576-4 (PMC8882295; doi:10.1186/s12889-022-12576-4)
Supplement: Supplementary file 3 — Additional file 3. Quality assessment of quantitative studies and quantitative component of mixed methods. [file 12889_2022_12576_MOESM3_ESM.docx]

**Additional file 3: Quality assessment of quantitative studies and quantitative component of mixed methods**

| Criteria and  Score | Question /objective clearly stated? | Design evident and appropriate to answer study question? | Method of subject selection described and appropriate | Subject characteristic sufficiently described? | Outcome measure well defined and robust to measure/  Mis-classification bias? Means of assessment reported? | Sample size appropriate? | Analytic methods described/  justified and appropriate? | Some estimate of variance reported for the main results? | Controlled for confounding? | Results reported in sufficient detail? | Conclusion supported by the results? | Ethics | Score |
| --- | --- | --- | --- | --- | --- | --- | --- | --- | --- | --- | --- | --- | --- |
| Banke Thomas et al, 2019 | Yes | Yes | Yes | Yes | Yes | Yes | Yes | Yes | Yes | Yes | Yes | Yes | **1.0** |
| Barnes et al, 2004 | Yes | Yes | Yes | Yes | Yes | Yes | Partially | Partially | No | Yes | Partially | Yes | **0.77** |
| Haworth et al, 2014 | Yes | Yes | Partially | Partially | Partially | Partially | Partially | No | No | Partially | Yes | Yes | **0.64** |
| Kisindja et al, 2017 | Yes | Yes | Yes | Yes | Yes | Yes | Partially | No | No | Yes | Partially | Yes | **0.73** |
| Lofters et al 2011 | Yes | Yes | Yes | Yes | Yes | Yes | Yes | Yes | Yes | Yes | Yes | No | **1.0** |
| Morrison, V 2000 | Yes | Yes | Yes | Yes | Partially | Partially | Partially | Partially | No | Partially | Partially | No | **0.64** |
| Odunukan et al 2015 | Partially | Yes | Yes | Yes | Yes | Partially | Yes | Yes | No | Partially | Yes | Partially | **0.77** |
| Pierce, H 2019 | Partially | Yes | Yes | Yes | Yes | Yes | Yes | Partially | Yes | Yes | Yes | No | **0.91** |
| Raben et al, 2018 | Yes | Yes | Yes | Yes | Yes | Partially | Yes | Yes | No | Yes | Yes | Yes | **0.86** |
| Raheel et al, 2012 | Yes | Yes | Yes | Yes | Yes | Yes | Yes | Yes | Yes | Yes | Yes | Yes | **1.0** |
| Redwood-Campbell et al, 2008 | Partially | Partially | Partially | Yes | Yes | Partially | Partially | No | No | Partially | Partially | Yes | **0.50** |
| Tanabe et al, 2017 | Yes | Yes | Yes | Yes | Yes | Yes | Yes | Yes | Yes | Yes | Yes | Yes | **1.0** |
