# Supplementary material for: Access to preventive sexual and reproductive health care for women from refugee-like backgrounds: a systematic review
Source: BMC Public Health. 2022 Feb 27;22:403. doi: 10.1186/s12889-022-12576-4 (PMC8882295; doi:10.1186/s12889-022-12576-4)
Supplement: Supplementary file 4 — Additional file 4. Quality assessment of qualitative studies and quality component of mixed methods. [file 12889_2022_12576_MOESM4_ESM.docx]

**Additional file 4: Quality assessment of qualitative studies and quality component of mixed methods**

| Criteria and  Score | Question / objective clearly described? | Design evident and appropriate to answer study question? | Context for the study is clear? | Connection to a theoretical framework / wider body of knowledge? | Sampling strategy described, relevant and justified? | Data collection methods clearly described and systematic? | Data analysis clearly described, complete and systematic? | Use of verification procedure(s) to establish credibility of the study? | Conclusion supported by the results? | Reflexivity of the account? | Ethics | Score |
| --- | --- | --- | --- | --- | --- | --- | --- | --- | --- | --- | --- | --- |
| Agbemenu et al, 2018 | Yes | Yes | Yes | Yes | Partially | Yes | Yes | Partially | Yes | Partially | No | **0.85** |
| Allen et al, 2019 | Yes | Yes | Yes | Yes | Partially | Yes | Yes | Yes | Yes | No | Yes | **0.85** |
| Babatunde-Sowole et al, 2020 | Yes | Partially | Yes | Yes | Yes | Partially | Yes | Yes | Yes | Partially | Partially | **0.85** |
| Cherri et al, 2017 | Yes | Yes | Yes | Yes | Partially | Yes | Partially | No | Yes | No | No | **0.70** |
| Ghebreyesus et al, 2020 | Yes | Yes | Yes | Yes | Yes | Yes | Yes | Yes | Yes | No | Yes | **0.90** |
| Gurnah et al, 2011 | Partially | Yes | Partially | Yes | Yes | Partially | Yes | No | Yes | No | Yes | **0.65** |
| Haworth et al, 2014 | Yes | Yes | Yes | Yes | Yes | Yes | Partially | No | Yes | No | Yes | **0.75** |
| Kabakkian-Khasholian et al, 2017 | Yes | Yes | Yes | Yes | Yes | Yes | Yes | Yes | Yes | Partially | Yes | **0.95** |
| Kim et al, 2017 | Yes | Yes | Yes | Yes | Yes | Yes | Yes | Yes | Yes | No | Yes | **0.90** |
| Lor et al, 2018 | Yes | Yes | Yes | Yes | Partially | Yes | Yes | Yes | Yes | Partially | Yes | **0.90** |
| Morrison, V 2000 | Yes | Yes | Yes | Yes | Yes | Yes | Partially | No | Yes | No | Yes | **0.75** |
| Parajuli et al, 2019 | Yes | Yes | Yes | Yes | Yes | Yes | Yes | Partially | Yes | Partially | Yes | **0.90** |
| Ross- Perfetti et al, 2019 | Yes | Yes | Yes | Yes | Yes | Yes | Yes | Yes | Yes | Yes | Yes | **1.0** |
| Royer et al, 2020 | Partially | Yes | Yes | Yes | Yes | Yes | Yes | Yes | Yes | No | Yes | **0.85** |
| Saadi et al, 2015 | Yes | Yes | Yes | Yes | Yes | Yes | Yes | Yes | Yes | Yes | No | **1.0** |
| Tanabe et al, 2017 | Yes | Yes | Yes | Yes | Partially | Yes | No | No | Partially | No | Yes | **0.60** |
| West et al, 2017 | Yes | Yes | Yes | Yes | Partially | Yes | Partially | No | Yes | No | Yes | **0.70** |
| Zhang et al, 2020 | Partially | Yes | Yes | Partially | Yes | Yes | Yes | Yes | Yes | Partially | Yes | **0.90** |
| Zhang et al, 2017 | Yes | Yes | Yes | Yes | Yes | Yes | Yes | Yes | Yes | No | Yes | **0.90** |
